# Construction of a geographically weighted nonparametric regression model fit test

**DOI:** 10.1016/j.mex.2023.102536

**Published:** 2024-01-02

**Authors:** Lilis Laome, I Nyoman Budiantara, Vita Ratnasari

**Affiliations:** a Institut Teknologi Sepuluh Nopember, Surabaya 60111 Indonesia; b Universitas Halu Oleo, Kendari 93121 Indonesia

**Keywords:** GWNR, Goodness of fit test, Poverty, Infant Death, Geographically Weighted Nonparametric Regression (GWNR)

## Abstract

One of the approach of Geographically Weighted Regression (GWR) models is the Geographically Weighted Nonparametric Regression (GWNR) has more parameters than the GWR model. Models with more parameters usually have better match values, which is an advantage, while models with fewer parameters have the advantage of being easier to use and interpret. However, a model with more parameters should be used if it is proven to be significantly superior. Therefore, the purpose of this study was to develop a hypothesis test of goodness of fit test for GWNR model. The goodness of fit test was performed for the real data. We found that the GWNR model was more suitable than the mixed nonparametric regression model. Some highlights of the proposed method are:

• A new model for GWR to overcome the unknown regression function by using mixed estimator spline truncated and fourier series at nonparametric regression
• Goodness of fit for GWNR to testing the model fit between the mixed nonparametric regression model and GWNR
• Applied goodness of fit test to poverty data in Sulawesi Island and infant mortality in East Java

## Graphical abstract

**Figure:**
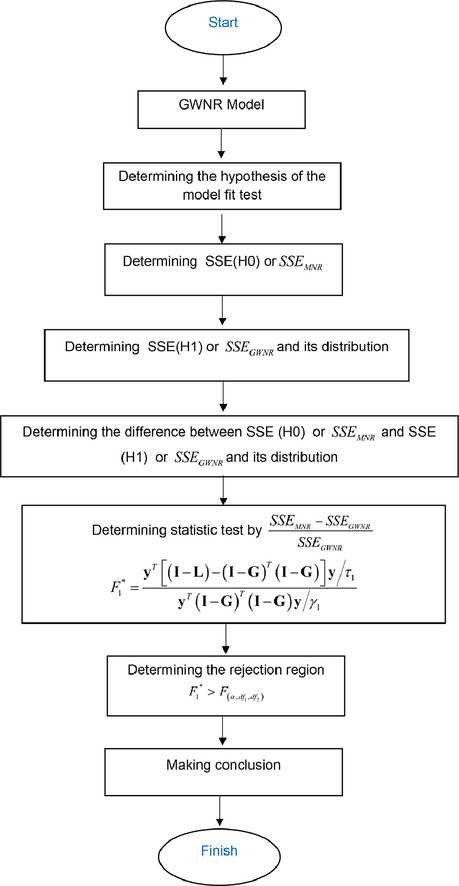

Specifications table

**Table:** 

Subject area:	Mathematics and Statistics
More specific subject area:	Statistics; Nonparametric Regression, Spatial Regression
Name of your method:	Geographically Weighted Nonparametric Regression (GWNR)
Name and reference of original method:	Original Method References: • Laome, L., Budiantara, I.N., dan Ratnasari, V. (2023), ``Estimation Curve of Mixed Spline Truncated and Fourier Series Estimator for Geographically Weighted Nonparametric Regression'', Mathematics 11(1), 152. • Leung, Y., Mei, CL., & Zhang, WX., 2000. Statistical Tests for Spatial Nonstationarity based on The Geographically Weighted Regression Model. Environment and Planning A. 32: 9–32.
Resource availability:	• Poor residents (Y) and the predictor (X) from Badan Pusat Statistik (BPS) in each Province in Sulawesi, Indonesia. • Infant death (Y) and the predictor (X) from Azizah (2013).

**Method details**

## Introduction

One popular spatial data analysis technique is the Geographically Weighted Regression (GWR) method, which has a wide range of applications. However, many researchers have further developed this model. As an illustration, [1] developed GWR on the negative binomial distribution, [2] developed GWR with GWR Multiscale, [3], [4], [5] discussed GWR semiparametric model, [6] built GWPolR (Geographically Weighted Polynomial Regression) model fit test, but GWR developed previously, still limited to parameterized regression. Based on Eubank [7], said that nonparametric regression is more flexible than parametric regression because nonparametric regression does not make assumptions about the shape of the function that connects the independent and dependent variables, but adjusts to the actual data. Research on nonparametric regression both single estimator [8], [9], [10] and mixed estimator [11], [12], [13] has been widely done. Furthermore, nonparametric regression on spatial data will be developed including: [14], [15], [16] studied a new method on GWR Spline Truncated model, and [17] developed GWR on spatial data.

Several references, such as [18] and [19], have shown that the GWR model can be expressed as follows:

(1) $$y_{i}=\beta_{0}\left(u_{i},v_{i}\right)+\sum_{k=1}^{K}\beta_{k}\left(u_{i},v_{i}\right)x_{ki}+\varepsilon_{i},\;i=1,2,...,n,\;k=1,2,...,K$$

where $\left(y_{i},x_{1i},...,x_{ki}\right)$ is the observed value of the response variable y and the predictor variables $x_{1}$, $x_{2}$, …, and $x_{k}$ at location $\left(u_{i},v_{i}\right)$, $\beta_{k}\left(u_{i},v_{i}\right)$ for *k* = 1,2,…,K is the parameter or regression coefficient at location $\left(u_{i},v_{i}\right)$ and $\varepsilon_{i}$, i=1,2,...,n, is the model error assumed to follow the Normal distribution with a mean 0 and variance is usually expressed by $\varepsilon_{i}∼N\left(0,\sigma^{2}\right)$.

The GWR is a modelling technique with regression coefficients or parameters that vary by location. However, when looking at the model in each location, all predictor variables are related to the response using a linear function. In fact, not all predictor variables have a linear relationship with the response. The most likely cause is that some of the predictor variables involved in the model have a non-linear pattern or do not have a certain pattern. To overcome this issue, [17] have made an extension or developed a model using a mixed nonparametric regression approach. In [17], the model is called Geographically Weighted Nonparametric Regression (GWNR).

The GWNR model is a development of the GWR model using a nonparametric regression function. The purpose of using this nonparametric regression function is to better accommodate the behavior of samples which may have one or more predictor variables that have an unknown relationship with the response [20], [21], [22]. Consequently, the GWNR model has more parameters than the GWR model. Generally, the advantage of a model with more parameters is that it has a higher fit value, while models with fewer parameters have the advantage of ease of application and interpretation. However, if the model with more parameters is found to be significantly better than that with fewer parameters, it should be used. With regards to the above mentioned background, it is necessary to discuss the significance test of GWNR model fit, and thus the purpose of this paper is to construct a hypothesis test of GWNR model fit.

**Model specifications and goodness of fit test procedures**

*Geographically Weighted Nonparametric Regression Model*

According to [17], the GWNR model is an extension of the GWR model in Eq. (1) which can be expressed as

(2) $$y_{i}=\sum_{p=0}^{P}f_{p}\left(x_{pi}\right)+\sum_{q=0}^{Q}g_{q}\left(z_{qi}\right)+\varepsilon_{i}\;\;i=1,2,...,n$$

(3) $$\begin{array}{cc} y & =X\beta \left(u_{i},v_{i}\right)+\mathrm{Za}\left(u_{i},v_{i}\right)+ɛ\\ & =\left[\begin{array}{cc} X & Z \end{array}\right]\left[\begin{array}{c} \beta \left(u_{i},v_{i}\right)\\ a\left(u_{i},v_{i}\right) \end{array}\right]+ɛ\\ & =Q^{*}\eta \left(u_{i},v_{i}\right)+ɛ \end{array}$$

where, vector y,ɛ of size n×1, matrix X of size n×(P(M+R)+1), matrix Z of size n×(Q(1+H), vector β has a size of (P(M+R)+1)×1, and vector a has a size of (Q(1+H)×1, $Q^{*}$ is a matrix of size n×(2+P(M+R)+Q(1+H)) and $\eta (u_{i},v_{i})$ is a vector of size (2+P(M+R)+Q(1+H))×1.

The WMLE estimator for the GWNR model parameters at location for $\left(u_{i},v_{i}\right)$ in Eq. (3) can be expressed as:

(4) $$\hat{\eta }\left(u_{i},v_{i}\right)={\left(Q^{*T}W\left(u_{i},v_{i}\right)Q^{*}\right)}^{-1}Q^{*T}W\left(u_{i},v_{i}\right)y,\;i=1,2,...,n$$

Based on Eq. (4), the estimated value vector of GWNR model for response variable **y** in n locations can be expressed as

(5) $${\hat{y}}_{GWNR}=\mathrm{Gy}$$

with

$$G=\left[\begin{array}{c} q_{1}^{T}{\left[Q^{*T}W\left(u_{1},v_{1}\right)Q^{*}\right]}^{-1}Q^{*T}W\left(u_{1},v_{1}\right)\\ q_{2}^{T}{\left[Q^{*T}W\left(u_{2},v_{2}\right)Q^{*}\right]}^{-1}Q^{*T}W\left(u_{2},v_{2}\right)\\ \vdots \\ q_{n}^{T}{\left[Q^{*T}W\left(u_{n},v_{n}\right)Q^{*}\right]}^{-1}Q^{*T}W\left(u_{n},v_{n}\right) \end{array}\right]$$

It is called the hat matrix of the GWNR model. Based on Eq. (5), the residual vector can be written as

(6) $${\hat{\varepsilon }}_{GWNR}=y-{\hat{y}}_{GWNR}=\left(I-G\right)y$$

Furthermore, with Eq. (6) the Sum of Squared Error (SSE) of the GWNR model is

(7) $$SSE_{GWNR}={\hat{\varepsilon }}_{GWNR}^{T}{\hat{\varepsilon }}_{GWNR}=y^{T}{\left(I-G\right)}^{T}\left(I-G\right)y$$

where **I** is the nth-order identity matrix.

*GWNR Model Fit Test*

In this section, GWNR model fit test statistics based on the SSE model will be constructed. The approximation distribution of the test statistic will then be investigated to test whether the GWNR model can describe the sample data significantly better than the GWR model. In this discussion, two assumptions are given as follows:

Assumption 1 the model error is $\varepsilon_{1}$, $\varepsilon_{2}$, …, $\varepsilon_{n}$ is assumed to be Normally distributed with a mean 0 and variance $\sigma^{2}$

Assumption 2 Suppose that $\hat{y}$ is the estimated value of y at location of $\left(u_{i},v_{i}\right)$. For $\hat{y}$ is unbiased estimator from E(y), that is $E\left(\hat{y}\right)=E\left(y\right)$

*Distribution of Sum of Squares Error of GWNR Model*

Suppose $q_{i}^{T}=(1\;\;x_{1i}\;\;...\;\;\;x_{1i}^{M}\;\;\;{\left(x_{1i}^{M}-t_{11}\right)}_{+}^{M}\;...\;\;\;{\left(x_{1i}^{M}-t_{1R}\right)}_{+}^{M}\;\;...\;\;\;\;x_{Pi}\;\;...\;\;\;x_{Pi}^{M}\;\;\;{\left(x_{Pi}^{M}-t_{P1}\right)}_{+}^{M}$ $...\;\;\;\;{\left(x_{Pi}^{M}-t_{PR}\right)}_{+}^{M}\;\;\frac{1}{2}\;\;z_{1i}\;\;\cos\left(z_{1i}\right)\;\;...\;\;\cos\left(Hz_{1i}\right)\;\;...\;\;z_{Qi}\;\;\cos\left(z_{Qi}\right)\;\;\cos\left(Hz_{Qi}\right))$ is the *i* th row of the matrix $Q^{*}$, i=1,2,...,n and $\hat{\eta }\left(u_{i},v_{i}\right)$ is the vector of parameter estimators at location $\left(u_{i},v_{i}\right)$. Estimated value for $y_{i}$ can be expressed as

(8) $$\begin{array}{cc} {\hat{y}}_{i} & =q_{i}^{T}\hat{\eta }\left(u_{i},v_{i}\right)\\ & =q_{i}^{T}{\left(Q^{*T}W\left(u_{i},v_{i}\right)Q^{*}\right)}^{-1}Q^{*T}W\left(u_{i},v_{i}\right)y \end{array}$$

and the residual model as ${\hat{\varepsilon }}_{i}=y_{i}-{\hat{y}}_{i}$, i=1,2,...,n.

For example ${\hat{y}}_{GWNR}={\left({\hat{y}}_{1}\;\;\;{\hat{y}}_{2}\;\;...\;\;{\hat{y}}_{n}\right)}^{T}$ denotes the vector of expected values for $y_{i}$ and

${\hat{\varepsilon }}_{GWNR}={\left({\hat{\varepsilon }}_{1}\;\;{\hat{\varepsilon }}_{2}\;\;...\;\;{\hat{\varepsilon }}_{n}\right)}^{T}$ states the residual vector, then

${\hat{y}}_{GWNR}=\mathrm{Gy}$ and ${\hat{\varepsilon }}_{GWNR}=y-{\hat{y}}_{GWNR}=\left(I-G\right)y$ as in Eq. (5) and (6), with **I** is the identity matrix of order n.

Suppose the sum of squared errors of GWNR model is denoted by $SSE_{GWNR}$ then

(9) $$SSE_{GWNR}=\sum_{i=1}^{n}{\hat{\varepsilon }}_{i}^{2}={\hat{\varepsilon }}_{GWNR}^{T}{\hat{\varepsilon }}_{GWNR}=y^{T}{\left(I-G\right)}^{T}\left(I-G\right)y$$

Furthermore, the unbiased estimator for the error variance is given in Theorem 1 below.

Theorem 1 *Suppose the GWNR model satisfies*Assumption 1*and*Assumption 2*, and*$\hat{\eta }\left(u_{i},v_{i}\right)$*as the parameter estimator at location*$\left(u_{i},v_{i}\right)$*, then the unbiased estimator for the error variance*$\left(\sigma^{2}\right)$*is given as follows:*

(10) $${\hat{\sigma }}^{2}=\frac{SSE_{GWNR}}{\gamma_{1}}$$

with $\gamma_{1}=tr\left({\left(I-G\right)}^{T}\left(I-G\right)\right)=n-\left(2tr\left(G\right)-tr\left(G^{T}G\right)\right)$

Proof Based on Assumption 1 and Assumption 2, then

$$\begin{array}{cc} E\left({\hat{\varepsilon }}_{GWNR}\right) & =E\left(y-{\hat{y}}_{GWNR}\right)\\ & =E\left(y\right)-E\left({\hat{y}}_{GWNR}\right)=0\\ E\left(ɛ{ɛ}^{T}\right) & =\sigma^{2}I \end{array}$$

where $ɛ={\left(\varepsilon_{1}\;\;\varepsilon_{2}\;\;...\;\;\varepsilon_{n}\right)}^{T}$ is the model error vector. Thus it can be expressed as follows:

$$\begin{array}{cc} SSE_{GWNR} & ={\left({\hat{\varepsilon }}_{GWNR}-E\left({\hat{\varepsilon }}_{GWNR}\right)\right)}^{T}\left({\hat{\varepsilon }}_{GWNR}-E\left({\hat{\varepsilon }}_{GWNR}\right)\right)\\ & ={ɛ}^{T}{\left(I-G\right)}^{T}\left(I-G\right)ɛ \end{array}$$

Therefore,

(11) $$\begin{array}{cc} E\left(SSE_{GWNR}\right) & =E\left({ɛ}^{T}{\left(I-G\right)}^{T}\left(I-G\right)ɛ\right)\\ & =E\left(tr\left({ɛ}^{T}{\left(I-G\right)}^{T}\left(I-G\right)ɛ\right)\right)\\ & =tr\left({\left(I-G\right)}^{T}\left(I-G\right)E\left(ɛ{ɛ}^{T}\right)\right)\\ & =\sigma^{2}\gamma_{1} \end{array}$$

where $\gamma_{1}=tr\left({\left(I-G\right)}^{T}\left(I-G\right)\right)$. Based on Eq. (11), the unbiased estimator for $\sigma^{2}$ is ${\hat{\sigma }}^{2}=\frac{SSE_{GWNR}}{\gamma_{1}}$. Operationally $\gamma_{1}$ can be expressed with the following formula

(12) $$\begin{array}{cc} \gamma_{1} & =tr\left({\left(I-G\right)}^{T}\left(I-G\right)\right)\\ & =tr\left(I\right)-tr\left(G\right)-tr\left(G^{T}\right)+tr\left(G^{T}G\right)\\ & =n-\left(2tr\left(G\right)+tr\left(G^{T}G\right)\right) \end{array}$$

Based on Theorem 1, the magnitude $SSE_{GWNR}$ can be used to estimate the error variance $\sigma^{2}$. This magnitude can be used to measure the suitability of the GWNR model for the sample data. The smaller the value of this quantity, the more appropriate the model is applied to the sample data. However, for the purpose of model fit testing, knowledge of the distribution of the magnitude requires $SSE_{GWNR}$. Therefore, a distribution approach is given for $SSE_{GWNR}$ which can be seen in Theorem 2 below.

Theorem 2 *Suppose the GWNR model meets*Assumptions 1*and*2*,*$\hat{\eta }\left(u_{i},v_{i}\right)$*is the model parameter estimator at location*$\left(u_{i},v_{i}\right)$*. Distribution for random variables*

(13) $${\hat{\sigma }}^{2}=\frac{\gamma_{1}SSE_{GWNR}}{\gamma_{2}\sigma^{2}}$$

can be approximated using the distribution $\chi_{r}^{2}$ with degrees of freedom $\;r=\frac{\gamma_{1}^{2}}{\gamma_{2}}$ with

(14) $$\gamma_{i}=tr\left({\left({\left(I-G\right)}^{T}\left(I-G\right)\right)}^{i}\right),\;i=1,2,$$

Proof From Eq. (18) it can be seen that$SSE_{GWNR}$ can be expressed as the square form of the Normal variable with ${\left(I-G\right)}^{T}\left(I-G\right)$ is a symmetry matrix and positive semidefinite. Based on the distribution theory of the quadratic form (1), it is known that a quadratic form of the Standard Normal variable, namely $\xi^{T}A\xi$ with ξ∼N(0,I) and A symmetry matrix, is distributed $\chi^{2}$ if and only if **A** is an idempotent matrix [7]. For variable

(15) $$\frac{SSE_{GWNR}}{\sigma^{2}}={\left(\frac{\varepsilon }{\sigma }\right)}^{T}{\left(I-G\right)}^{T}\left(I-G\right)\left(\frac{\varepsilon }{\sigma }\right)$$

it is known that $\frac{\varepsilon }{\sigma }∼N\left(0,I\right)$, but the matrix ${\left(I-G\right)}^{T}\left(I-G\right)$ is generally not idempotent, because its forming element is the weight matrix $W(u_{i},v_{i})$ which is different at each location *i.* As a result, $\frac{SSE_{GWNR}}{\sigma^{2}}$ is not exact $\chi^{2}$ distribution, but the distribution can be determined with the approach$\chi^{2}$. Based on the quadratic shape distribution theory [14], the distribution approach to the quadratic form (18) can be done by multiplying the variables $\chi_{r}^{2}$ by a constant c, which can be written by $c\chi_{r}^{2}$ . Furthermore, *c* and *r* are determined so that the mean and variance of $c\chi_{r}^{2}$, the approximated quadratic $c\chi_{r}^{2}$ form correspond to each other. For the variable $\chi_{r}^{2}$, based on the theory, the mean and variance are r and 2r, respectively. Therefore, the mean and variance of the variable are *cr* and 2*c^2^r*, respectively.

For quadratic form variables $\frac{SSE_{GWNR}}{\sigma^{2}}$, based on Eq. (11) is known to have a mean $\gamma_{1}$. The variance is described below. Since the matrix ${\left(I-G\right)}^{T}\left(I-G\right)$ is symmetry and positive semidefinite, there exists an orthogonal matrix **P** of order *n* such that

(16) $$P^{T}{\left(I-G\right)}^{T}\left(I-G\right)P=\Lambda =diag\left(\lambda_{1},\lambda_{2},...,\lambda_{n}\right)$$

where Λ is a diagonal matrix whose main diagonal elements are the eigenvalues of the matrix ${\left(I-G\right)}^{T}\left(I-G\right)$, suppose that

(17) $$\zeta ={\left(\zeta_{1},\zeta_{2},...,\zeta_{n}\right)}^{T}=P^{T}\frac{\varepsilon }{\sigma }$$

According to the nature of multivariate Normal distribution, $\zeta_{1},\zeta_{2},...,\zeta_{n}$ is an independent and identical random variable with standard Normal distribution, which is written by $\zeta_{i}∼iid\;N\left(0,1\right)$. On the other hand, from Eq. (17), it is obtained $\frac{\varepsilon }{\sigma }=P\zeta$, and the line from Eq. (15) that gets

(18) $$\begin{array}{ccc} \frac{SSE_{GWNR}}{\sigma^{2}} & = & \zeta^{T}P^{T}{\left(I-G\right)}^{T}\left(I-G\right)P\zeta \\ & = & \zeta^{T}\Lambda \zeta \\ & = & \sum_{i=1}^{n}\lambda_{i}\zeta_{i}^{2} \end{array}$$

In accordance with the distribution theory, because $\zeta_{i}∼iid\;N\left(0,1\right)$ then $\zeta_{i}^{2}∼iid\;\chi_{\left(1\right)}^{2}$ for i=1,2,...,n, therefore $\text{var}(\zeta_{i}^{2})=2$ and

(19) $$\text{var}\left(\frac{SSE_{GWNR}}{\sigma^{2}}\right)=\sum_{i=1}^{n}\lambda_{i}^{2}\text{var}\left(\zeta_{i}^{2}\right)=2\sum_{i=1}^{n}\lambda_{i}^{2}$$

If $\lambda_{1},\lambda_{2},...,\lambda_{n}$ are the eigenvalues of the matrix${\left(I-G\right)}^{T}\left(I-G\right)$, then $\lambda_{1}^{2},\lambda_{2}^{2},...,\lambda_{n}^{2}$ is the eigenvalue of the matrix ${\left({\left(I-G\right)}^{T}\left(I-G\right)\right)}^{2}$. As a result,

(20) $$\begin{array}{cc} \text{var}\left(\frac{SSE_{GWNR}}{\sigma^{2}}\right) & =2tr\left({\left({\left(I-G\right)}^{T}\left(I-G\right)\right)}^{2}\right)\\ & =2\gamma_{2} \end{array}$$

where $\gamma_{2}=tr\left({\left({\left(I-G\right)}^{T}\left(I-G\right)\right)}^{2}\right)$

Based on Eqs. (11) and (20), the following system of equations can be formed

(21) $$\begin{array}{c} cr=\gamma_{1}\\ 2c^{2}r=2\gamma_{2} \end{array}$$

The solution of the system of Eqs. (21) is $c=\frac{\gamma_{2}}{\gamma_{1}}$ and $r=\frac{\gamma_{1}^{2}}{\gamma_{2}}$. Thus, the distribution of $\frac{SSE_{GWNR}}{c\sigma^{2}}$ or $\frac{\gamma_{1}SSE_{GWNR}}{\gamma_{2}c\sigma^{2}}$ can be approximated using the distribution $\chi^{2}$ with degrees of freedom $r=\frac{\gamma_{1}^{2}}{\gamma_{2}}$ where $\gamma_{i}=tr\left({\left({\left(I-G\right)}^{T}\left(I-G\right)\right)}^{i}\right)$, i=1,2.

*Test Statistics of GWNR Model Fit and Distribution*

The Sum of Squared Error and its approximation distribution that have been obtained in the Theorem 2 will then be used to test the model fit, to determine whether GWNR can model the data significantly better than MNR. In this model fit test, it is hypothesized as follows:

(22) $$\begin{array}{cc} H_{0}: & \eta_{j}\left(u_{i},v_{i}\right)=\eta_{j}\;;\;j=1,2,...,J\;;\;i=1,2,...,n\\ & \left(\text{GWNR}\;\text{model}\;\text{is}\;\text{not}\;\text{different}\;\text{from}\;\text{MNR}\;\text{model}\right)\\ H_{1}: & \text{there}\;\text{is}\;\text{at}\;\text{least}\;\text{one}\;\;\eta_{j}\left(u_{i},v_{i}\right)\neq \eta_{j}\;\\ & \left(\text{GWNR}\;\text{model}\;\text{is}\;\text{significantly}\;\text{better}\;\text{fit}\;\text{than}\;\text{MNR}\;\text{model}\right) \end{array}$$

To test the above hypothesis, this paper constructs a test statistic and its approximation distribution through Theorem 3 below.

Theorem 3 *Suppose* $SSE_{MNR}=y^{T}{\left(I-L\right)}^{T}\left(I-L\right)y$ *is the sum of squared errors of MNR model where* ***L*** *is the hat matrix of MNR model, and* $SSE_{GWNR}=y^{T}{\left(I-G\right)}^{T}\left(I-G\right)y$ *is the sum of squared errors of GWNR model where* ***G*** *is the hat matrix of GWNR model. Distribution of statistics*

(23) $$F_{1}^{*}=\frac{y^{T}\left[\left(I-L\right)-{\left(I-G\right)}^{T}\left(I-G\right)\right]y/\tau_{1}}{y^{T}{\left(I-G\right)}^{T}\left(I-G\right)y/\gamma_{1}}$$

Approximates the *F* distribution with numerator degrees of freedom $df_{1}=\frac{\tau_{1}^{2}}{\tau_{2}}$ and denominator degrees of freedom $df_{2}=\frac{\gamma_{1}^{2}}{\gamma_{2}}$ with $\tau_{i}=tr\left({\left(\left(I-L\right)-{\left(I-G\right)}^{T}\left(I-G\right)\right)}^{i}\right)$ and $\gamma_{i}=tr\left({\left({\left(I-G\right)}^{T}\left(I-G\right)\right)}^{i}\right)$ for i=1,2.

Proof If the GWNR model is used to model the sample data, then the sum of squared errors can be expressed by $SSE_{GWNR}=y^{T}{\left(I-G\right)}^{T}\left(I-G\right)y$. Based on Theorem 2, the distribution of $\frac{\gamma_{1}SSE_{GWNR}}{\gamma_{2}\sigma^{2}}$ can be approximated by the distribution $\chi_{r}^{2}$ with degrees of freedom $r=\frac{\gamma_{1}^{2}}{\gamma_{2}}$, for

(24) $$\gamma_{i}=tr\left({\left({\left(I-G\right)}^{T}\left(I-G\right)\right)}^{i}\right),\;i=1,2$$

If the MNR model is used to model the sample data, then the sum of squared errors can be expressed by $SSE_{MNR}=y^{T}{\left(I-L\right)}^{T}\left(I-L\right)y$ where **L** is the hat matrix of the MNR model. According to [19], the distribution of the variable $\frac{\tau_{1}\left(SSE_{MNR}-SSE_{GWNR}\right)}{\tau_{2}\sigma^{2}}$ can be approximated by the distribution $\chi_{d}^{2}$ with degrees of freedom $d=\frac{\tau_{1}^{2}}{\tau_{2}}$ for

(25) $$\tau_{i}=tr\left({\left(\left(I-L\right)-{\left(I-G\right)}^{T}\left(I-G\right)\right)}^{i}\right)$$

In the distribution theory, the variable $\frac{\chi_{r}^{2}/\tau_{1}}{\chi_{d}^{2}/\gamma_{1}}$ is *F*-distributed with independent degrees of the numerator r and independent degrees of the denominator d. Therefore, the distribution of the test statistic

(26) $$F_{1}^{*}=\frac{y^{T}\left[\left(I-L\right)-{\left(I-G\right)}^{T}\left(I-G\right)\right]y/\tau_{1}}{y^{T}{\left(I-G\right)}^{T}\left(I-G\right)y/\gamma_{1}}$$

Approximates the *F* distribution with independent degrees of numerator$\tau_{1}$ and independent degrees of denominator$\gamma_{1}$. If simplified, Eq. (26) becomes Eq. (23), where

$\tau_{i}=tr\left({\left(\left(I-L\right)-{\left(I-G\right)}^{T}\left(I-G\right)\right)}^{i}\right)$ and $\gamma_{i}=tr\left({\left({\left(I-G\right)}^{T}\left(I-G\right)\right)}^{i}\right)$ for i=1,2.

If $H_{0}$ is true, the magnitude $\frac{SSE_{MNR}-SSE_{GWNR}}{SSE_{GWNR}}$ is logically close to zero. If $H_{0}$ is false, the magnitude tends to be large close to 1. Based on this logic, the larger the $F_{1}^{*}$ value, the more supportive it is to reject $H_{0}$, which means that the GWNR model is significantly more suitable than the MNR model. Therefore, at a given significance α level, $H_{0}$ is rejected if $F_{1}^{*}>F_{\left(\alpha ,df_{1},df_{2}\right)}$ and it is concluded that the GWNR model is significantly better fit than the MNR model.

### Application of data

Two cases were used in this research: the infant mortality case in East Java, which affected 38 city districts, and the poverty case on Sulawesi Island, which affected 81 city districts. The following is explained using the steps from the research.

## Case of poor population

As a case example, this paper uses data on poor population of 81 districts/cities in Sulawesi Island, Indonesia in 2021. The data was obtained from [23], [24], [25], [26], [27], [28]. The variables involved in the model include: Percentage of Poor Population (Y), Firewood User Households (FUH) (X_1_), Households without Latrines (HWL) (X_2_), Households with Adequate Drinking Water Sources (HWADWS) (X_3_) and Average Employee Net Wage (AENW) (X_4_).

**Fig. 1:**
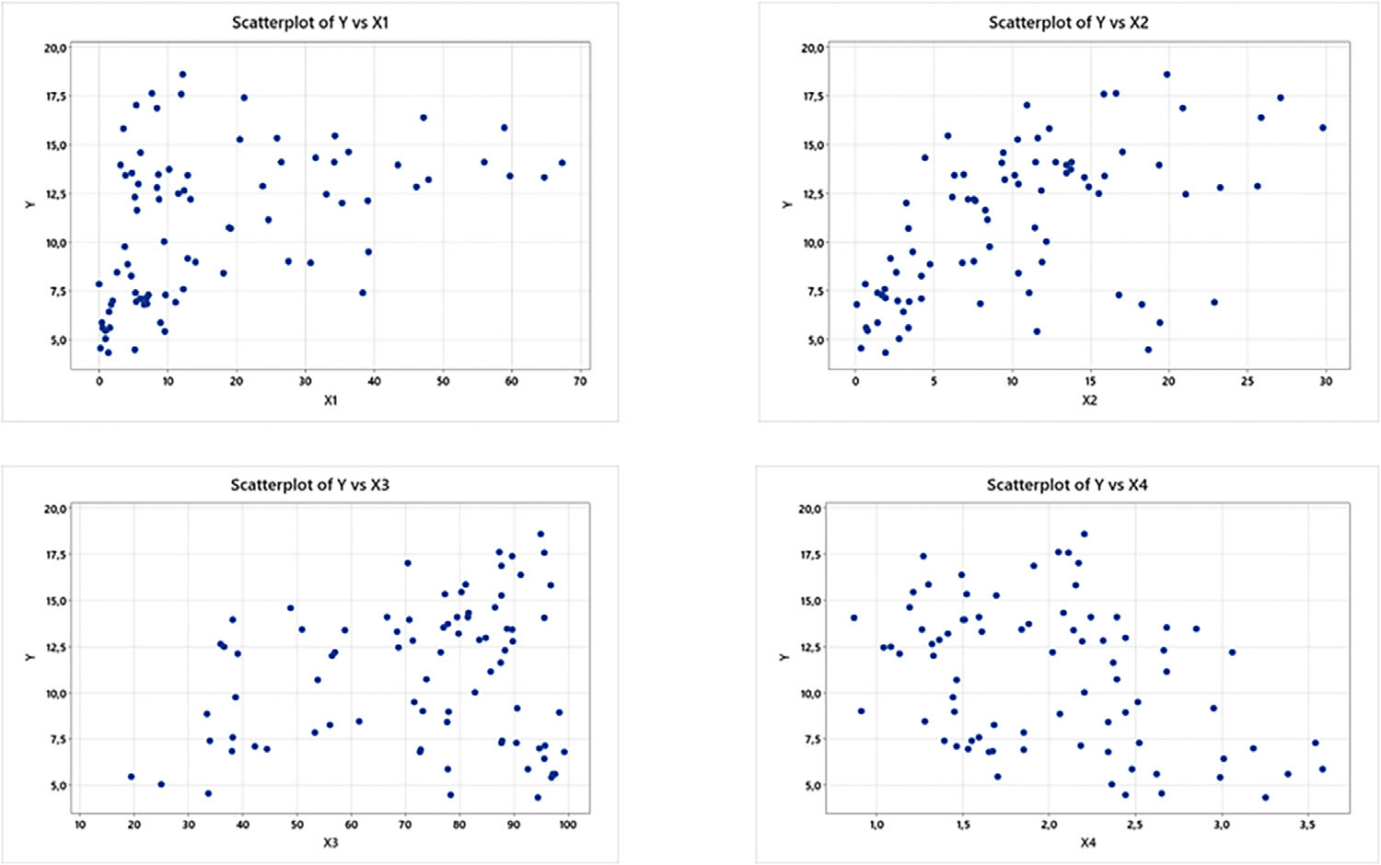
Scatter Plot between Response Variables and Predictor Variables for First Case.

The scatter plot for the four predictor variables X_1_, X_2_, X_3_, and X_4_ against the response variable (Y) can be seen in Fig. 1. Visually, it can be seen that the relationship between the predictor variables and the response variable shows an unknown relationship pattern. One alternative that can be used is to model the data with a nonparametric regression model. In this study, we will use a mixed estimator in the GWNR model, namely a mixed estimator of truncated spline and fourier series. Furthermore, MNR and GWNR models are given as in Eqs. (27), (28), and (29)

The Mixed Nonparametric Regression Model for first case is:

(27) $$\begin{array}{ccc} y & = & \beta_{0}+\beta_{11}x_{2}+\beta_{12}\left(x_{2}-t_{11}\right)+\beta_{21}x_{4}+\beta_{22}\left(x_{4}-t_{11}\right)+\frac{1}{2}\theta_{0}+\gamma_{1}z_{1}+\theta_{11}\cos\left(z_{1}\right)+\gamma_{2}z_{2}+\theta_{21}\cos\left(z_{2}\right)+\varepsilon \\ \hat{y} & = & -10,45+0,56x_{2}-0,55\left(x_{2}-8,19\right)+16,09x_{4}-18\left(x_{4}-1,12\right)-5,23+0,04x_{1}-0,02\cos\left(x_{1}\right)+0,05x_{3}+0,85\cos\left(x_{3}\right) \end{array}$$

The GWNR model for some locations to first case are:

For Kendari City:

(28) $$\begin{array}{ccc} {\hat{y}}_{40} & = & 0,68+0,44x_{2}-0,34{\left(x_{2}-8,19\right)}_{+}-3,04x_{4}-3,85{\left(x_{4}-1,12\right)}_{+}+0,34-\;0,19x_{1}+2,96\;\cos\left(x_{1}\right)+0,23x_{3}+1,004\cos\left(x_{3}\right) \end{array}$$

For Boalemo City

(29) $$\begin{array}{ccc} {\hat{y}}_{6} & = & 10,17+1,18x_{2}-0,89{\left(x_{2}-8,19\right)}_{+}-17,04x_{4}+13,02{\left(x_{4}-1,12\right)}_{+}+5,08-0,14x_{1}+1,53\cos\left(x_{1}\right)+0,16x_{3}+0,39\cos\left(x_{3}\right) \end{array}$$

Using the test statistic in Eq. (26), it is obtained the value $F_{1}^{*}=1.98$ and $F_{\left(0.05;53.85;37.49\right)}=1.67$, because the value of $F_{1}^{*}>F_{\left(0.05;53.85;37.49\right)}$ then reject H_0_, which shows the GWNR model is significantly more suitable than the MNR model. Thus the GWNR model can be applied in modeling the percentage of poor people in Sulawesi Island in 2021. Furthermore, a measure of model fit is given based on the smallest Mean Square Error (MSE) and highest R^2^ value, which is presented in Table 1.

**Table 1:** Best Model Selection for first case.

Model	MSE	R^2^ (%)
Multiple Linear Regression	9.13	37.42
Mixed Nonparametric Regression	7.16	50.28
GWR	3.73	70.25
GWNR	1.64*	86.98*

*Best Model.

Based on Table 1, it shows that the GWNR model with a mixed spline truncated and fourier series estimator is better modeled on the Sulawesi island poverty data based on the MSE value and R^2^ value.

## Infant death case

**Fig. 2:**
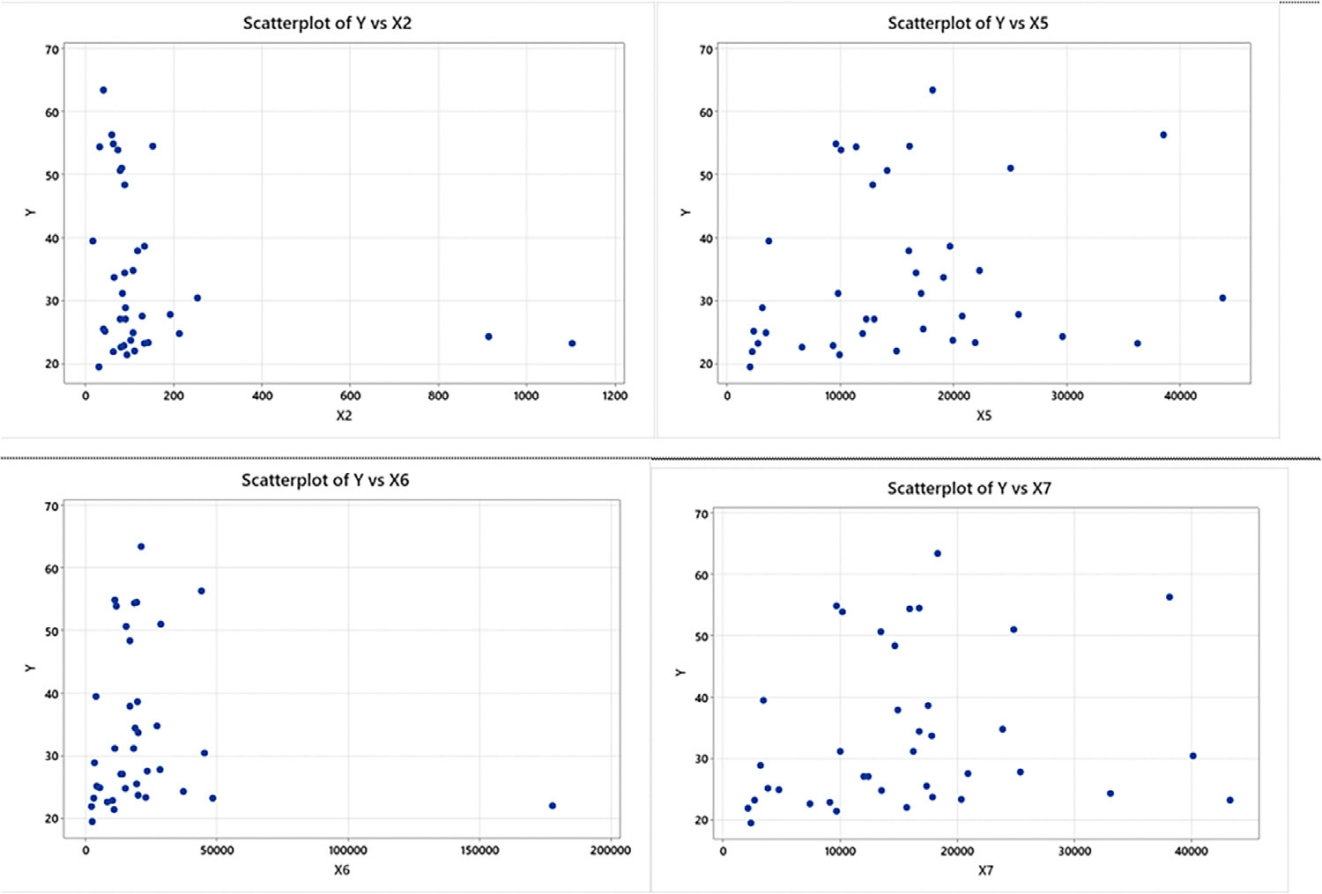
Scatter Plot between Response Variables and Predictor Variables for Second Case.

The following case is infant mortality in East Java for 38 city districts with research variables namely the number of infant deaths (Y), the number of medical personnel (X_1_), vitamin administration (X_2_), maternal health (X_3_) and infant health (X_4_) [29]. Like the research steps in the previous case, the first step is to create a scatter plot as shown in Fig. 2 below.

The Mixed Nonparametric Regression Model for second case is:

(30) $$\begin{array}{ccc} y & = & \beta_{0}+\beta_{11}x_{3}+\beta_{12}\left(x_{3}-t_{11}\right)+\beta_{21}x_{4}+\beta_{22}\left(x_{4}-t_{11}\right)+\frac{1}{2}\theta_{0}+\gamma_{1}z_{1}+\theta_{11}\cos\left(z_{1}\right)+\gamma_{2}z_{2}+\theta_{21}\cos\left(z_{2}\right)+\varepsilon \\ \hat{y} & = & -1.09-6.95x_{2}-4.67\left(x_{2}-46127\right)+1.05x_{4}-7.95\left(x_{4}-12389\right)-5.47-3.48x_{1}\\ & & 7.09\cos\left(x_{1}\right)-1.16x_{3}-1.03\cos\left(x_{3}\right) \end{array}$$

The GWNR model for some locations to second case are:

For Probolinggo City:

(31) $$\begin{array}{ccc} {\hat{y}}_{13} & = & -6.15\times 10^{-6}+0.04x_{3}-0.05{\left(x_{3}-46127.25\right)}_{+}-0.04x_{4}-0.003{\left(x_{4}-12389\right)}_{+}\\ & & -\;3.07\times 10^{-6}-\;0.004x_{1}+9.14\times 10^{-5}\;\cos\left(x_{1}\right)+0.002x_{2}+0.00019\cos\left(x_{2}\right) \end{array}$$

For Blitar City

(32) $$\begin{array}{ccc} {\hat{y}}_{31} & = & 0.02+0.34x_{3}+0.65{\left(x_{3}-46127.25\right)}_{+}-0.39x_{4}-0.06{\left(x_{4}-12389\right)}_{+}\\ & & +\;0.01+0.12x_{1}+0.005\;\cos\left(x_{1}\right)+0,01x_{2}-0.02\cos\left(x_{2}\right) \end{array}$$

Eq. (26), which employs the test statistic, yields the value $F_{1}^{*}=6.82$ and $F_{\left(0.05;27.88;11.15\right)}=2.56$, since the value of $F_{1}^{*}>F_{\left(0.05;27.88;11.15\right)}$, rejects H_0_, indicating that the GWNR model is substantially more appropriate than the MNR model. Consequently, the number of infant deaths in East Java in 2012 can be estimated using the GWNR model. Additionally, Table 2 presents a measure of model fit based on the lowest Mean Square Error (MSE) and highest R^2^ value.

**Table 2:** Best Model Selection for the second cases.

Model	MSE	R^2^ (%)
Multiple Linear Regression	117.5	34.32
Mixed Nonparametric Regression	126,55	59
GWR	32.16	82.94
GWNR	5.01*	97*

*Best Model.

Table 2 illustrates that, when analyzing the number of infant deaths data, the GWNR model featuring a mixed spline truncated and fourier series estimator performs better in terms of MSE and R^2^ values.

## Conclusion

The GWNR model fit test is constructed to determine whether the GWNR model describes a particular data set significantly better than MNR. This test can be constructed based on the sum of squared errors of the model. The GWNR model fit test statistic can be approximated using the F distribution. The use of the GWNR model fit test to examine the poor population data in Sulawesi and number of infant deaths shows that the GWNR model is better than the mixed nonparametric regression model in its application.

## Ethics statements

The data used in this research are secondary data derived from the official website of BPS Provinces in Sulawesi, Indonesia.

## CRediT authorship contribution statement

**Lilis Laome:** Conceptualization, Methodology, Writing – original draft. **I Nyoman Budiantara:** Conceptualization, Methodology, Writing – review & editing, Supervision. **Vita Ratnasari:** Methodology, Writing – review & editing, Supervision.

## Declaration of Competing Interest

The authors declare that they have no known competing financial interests or personal relationships that could have appeared to influence the work reported in this paper.

## Data Availability

The authors do not have permission to share data.
